# Symbiotic interactions and climate change implications of the octocoral microbiome

**DOI:** 10.1093/ismejo/wrag246

**Published:** 2026-09-15

**Authors:** Tina Keller-Costa, Romie Tignat-Perrier, Matilde Marques, Christine Ferrier-Pagès, Claudia Pogoreutz

**Affiliations:** Department of Bioengineering, Instituto Superior Técnico, Av. Rovisco Pais 1, 1049-001 Lisbon, Portugal; iBB—Institute for Bioengineering and Biosciences and i4HB—Institute for Health and Bioeconomy, Instituto Superior Técnico, Av. Rovisco Pais 1, 1049-001 Lisbon, Portugal; Equipe Biologie Corallienne, Centre Scientifique de Monaco, MC-98000 Principality of Monaco; Unité de Recherche sur la Biologie des Coraux Précieux CSM—CHANEL, Centre Scientifique de Monaco, MC-98000 Principality of Monaco; Department of Bioengineering, Instituto Superior Técnico, Av. Rovisco Pais 1, 1049-001 Lisbon, Portugal; iBB—Institute for Bioengineering and Biosciences and i4HB—Institute for Health and Bioeconomy, Instituto Superior Técnico, Av. Rovisco Pais 1, 1049-001 Lisbon, Portugal; Equipe Biologie Corallienne, Centre Scientifique de Monaco, MC-98000 Principality of Monaco; UAR 3278 CRIOBE, PSL Université Paris: EPHE-UPVD-CNRS, Université de Perpignan Via Domitia, 52 Avenue Paul Alduy, 66860, Perpignan Cedex, France

**Keywords:** holobiont, host–microbe interactions, symbioses, soft corals, gorgonians, *Endozoicomonadaceae*, *Spirochaetaceae*, nutrient cycling, pathobiome, ocean warming

## Abstract

Octocorals are vital components of tropical, temperate, and cold-water benthic marine ecosystems. Their associated microbiomes, comprising microeukaryotes, prokaryotes, and viruses, are increasingly recognized as central to host health, nutrient cycling, and chemical defense. Metagenomics and amplicon sequencing have uncovered taxonomic and functional complexity within these microbial communities, revealing patterns of host specificity and health status, along with seasonality and geographic structuring. However, anthropogenic stressors, particularly those associated with global climate change, exert intense pressure on coral-dominated ecosystems, leading to complex and poorly understood local and regional patterns of octocoral expansion and mortality. Microbial interactions may be a main driver of these contrasting outcomes by mediating the ecological resilience of octocorals to environmental stress. We synthesize the current state of research on the diversity, organization, and function of the octocoral microbiome, and identify critical knowledge gaps on octocoral holobionts relative to scleractinian corals. Our meta-analysis of 79 publicly available bacterial genomes from octocorals reveals group-specific specialization in denitrification and nitrate assimilation, along with widespread capacities for essential amino acid, cofactor, and vitamin production, suggesting important contributions to nutrient cycling in the holobiont. While sampling efforts between cultured and uncultured lineages are even, our genomic survey reveals strong sampling bias toward the Atlantic Ocean, temperate gorgonians, and healthy host states, whereas bacterial genomes representing the pathobiome, tropical and/or deep-sea regions, and other octocoral taxa remain underrepresented. Accordingly, we propose future research directions to advance understanding of octocoral microbiome ecology and its role in the resilience of tropical, temperate, and cold-water coral reefs.

## Introduction

Octocorals (class Octocorallia) are a morphologically and functionally diverse group of Anthozoa (phylum Cnidaria) that comprises two orders, Malacalcyonacea and Scleralcyonacea [1]. Malacalcyonacea includes sea fan- and sea whip-like gorgonians with proteinaceous axes, as well as soft corals, whereas Scleralcyonacea comprises sea pens, blue corals, and gorgonian lineages with predominantly calcareous axial skeletons, including the red coral *Corallium rubrum*. Octocorals differ from reef-building, scleractinian corals (order Scleractinia, class Hexacorallia), the primary ecosystem engineers of tropical coral reefs, in the radial symmetry of their polyps, skeletal composition, and life history strategies [2, 3]. Shallow tropical reefs in the Indo-Pacific and Caribbean harbor particularly high octocoral diversity; yet, these animals are also widespread in temperate, cold-water, and deep-sea environments, where they often dominate as benthic structuring organisms and foundational species of marine animal forests [4]. Octocorals have traditionally been underrepresented in coral reef research, being largely considered secondary contributors to the coral reef framework. Consequently, octocorals have only lately been recognized as important ecological players that contribute to habitat complexity and trophic networks. Recent biogeographic patterns reveal contrasting trajectories for octocoral communities under climate-driven ocean change. In some mesophotic and degraded reefs, octocorals now dominate over scleractinians [5]. In the tropical western Atlantic and Caribbean, for instance, they expanded and became the dominant benthic organisms, forming extensive octocoral forests that now underpin reef structure and ecosystem function. This proliferation is particularly evident in the U.S. Virgin Islands, Puerto Rico, the Florida Keys, and the southern Caribbean (Curaçao, Bonaire) [6]. The dominance of tropical octocorals is attributed to their comparatively higher resistance and resilience to stressors such as eutrophication, warming, and ocean acidification—the main drivers of global scleractinian coral decline [7–9]. In contrast, temperate octocoral populations, particularly in the Mediterranean, have suffered severe declines, as recurrent marine heatwaves have caused repeated mass mortalities in key habitat-forming species, threatening the ecological balance of octocoral-dominated marine forests [10–12]. Collectively, these divergent trends underscore octocorals’ sensitivity and adaptability to regional climatic regimes, highlighting their dual role as indicators and architects of shifting marine ecosystems.

Like all other cnidarians, octocorals are holobionts—complex organisms composed of the coral host and its associated microorganisms, including archaea, bacteria, viruses, and microeukaryotes such as microalgae, protozoans, and fungi [13]. The most extensively studied association within this consortium is that between octocorals and their intracellular photosynthetic dinoflagellates of the Symbiodiniaceae family [14], which provide the host with photosynthates in exchange for ammonium waste and respiratory carbon [15, 16]. Current research indicates that Symbiodiniaceae specificity in octocorals may be more selective and stable than previously thought [17], though some plasticity occurs along environmental gradients [18]. Although symbiosis with Symbiodiniaceae is common in shallow tropical and subtropical octocorals, most temperate, mesophotic, and deep-sea species lack photosymbionts and rely mostly on heterotrophy [19]. The broader octocoral microbiome, however, remains poorly understood compared with that of scleractinian corals, with the notable exception of studies on drug discovery [20–22]. Emerging evidence suggests that octocoral-associated microbial communities play key roles in nutrient cycling [17, 23, 24], chemical defense [20, 22, 25], and stress resistance [26]. Yet, their functional interactions, specificity, and stability under stress remain largely unknown. Most existing studies are descriptive, focusing on diversity rather than function. As some octocoral species expand in degraded habitats while others suffer mass mortality during heatwaves, a deeper understanding of their microbiome’s structure, function, and interactions is crucial to elucidate the microbial mechanisms driving their contrasting resilience and adaptability, and to better predict the future of reefs and marine forests under global change.

In this review, we synthesize current knowledge on the diversity, organization, and function of the octocoral microbiome with primary focus on the less explored prokaryotic and eukaryotic microbiota (see [13, 27, 28] for an excellent overview of octocoral-associated Symbiodiniaceae). We present a meta-analysis of all 79 publicly available (8 September 2025) octocoral-derived bacterial genomes obtained from 16 studies, detailing their taxonomy, phylogenomic relationships, provenance, and functional capacities related to nitrogen, carbon, and sulfur cycling, as well as amino acid, cofactor, vitamin, and secondary metabolite biosynthesis. Finally, we examine octocoral holobionts responses to environmental stressors, compare octocoral and scleractinian microbiome research, identify major knowledge gaps, and outline future research priorities to advance the field of octocoral microbiome science.

### Composition of the octocoral microbiome

While multiple studies have explored octocoral microbial diversity, integrating the existing knowledge remains challenging due to methodological heterogeneity among surveys. Differences in sample processing, deoxyribonucleic acid (DNA) extraction methods, primer selection, sequencing platforms, and bioinformatic pipelines [e.g. operational taxonomic unit (OTU)- versus amplicon sequence variant (ASV)-based approaches] can influence the inferred community composition. Most studies rely on DNA-based amplicon sequencing of the 16S ribosomal ribonucleic acid (rRNA) gene, which may not fully reflect the metabolically active microbiome. RNA-based approaches (e.g. using complementary DNA generated from rRNA transcripts) are rare and may introduce other biases related to extraction efficiency and RNA stability. Variation in sampling design, including differences in spatial or temporal scales can also affect comparability across studies. Although our review cannot explicitly account for all methodological differences, the patterns observed across studies still provide valuable insights into octocoral-associated microbial communities.

#### Bacteria and archaea

Bacterial communities associated with octocorals are diverse, uneven, species-specific, and exhibit high metabolic potential [13, 23, 29, 30]. Within the Malacalcyonacea, large fluctuations in bacterial community diversity have been reported, ranging from dozens [31] to thousands of phylotypes [32]. These communities are often largely dominated by *Pseudomonadota*, especially *Gammaproteobacteria* [13], with increases in *Flavobacteriia* and *Alphaproteobacteria* in necrotic tissues [29] (Fig. 1). *Endozoicomonadaceae* are particularly common in many octocoral species and environments [13, 29, 30, 33]. While high relative abundances of *Endozoicomonadaceae* and *Mycoplasmatales* are typical for gorgonians across ocean basins (Fig. 1a) [23, 29, 34, 35], other frequently reported octocoral-associated taxa include *Rhodobacterale*s, *Rhodospirillales*, *Hyphomicrobiales* [13, 29, 35–37], *Cellvibrionales*, unclassified *Oceanospirillales* [23, 29, 38], and *Spirochaetaceae* [13, 35, 37, 38]. These associations show variations at the octocoral species and family levels, and across depths and climatic conditions. Similar to scleractinian corals [39], a certain degree of co-phylogeny or co-diversification of *Endozoicomonadaceae* and other bacterial associates with their host has been suggested for octocorals in the Caribbean [40], Atlantic [29], Mediterranean, and the Red Sea [35]. However, with the exception of the work by Prioux *et al.* [35], assessments are of limited scope, and more comprehensive analyses of the evolutionary history between octocorals and their bacterial symbionts, using broader datasets and a greater taxonomic and geographic diversity of octocoral species, are needed to further test and validate co-phylogeny.

**Figure 1:**
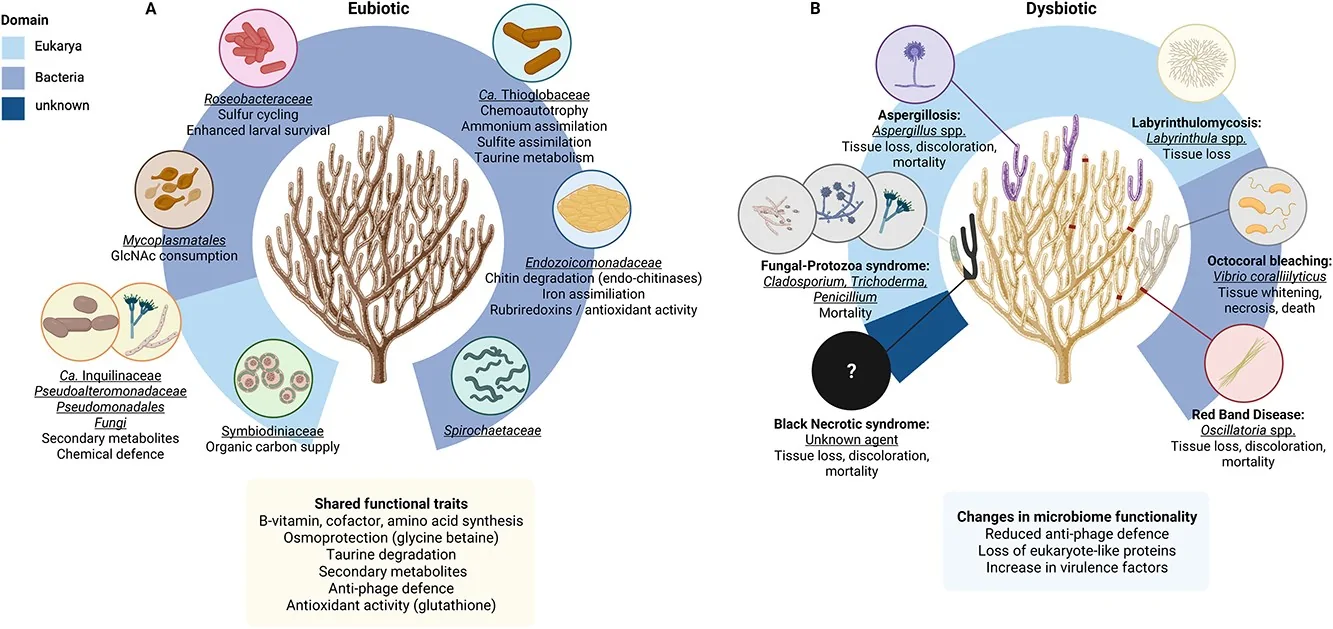
Taxonomic affiliation and proposed functions or traits of microorganisms frequently associated with the healthy octocoral holobiont (A), and reported octocoral pathogens, opportunists, diseases and induced phenotypes of the diseased octocoral holobiont (B). Figure created in BioRender. Pogoreutz, C. (2026) https://BioRender.com/dnkaoy0.

While studies of octocoral bacterial communities remain skewed toward Malacalcyonacea, microbiome data for the less studied Scleralcyonacea have also been increasing, particularly for the red coral *C. rubrum*, which is frequently dominated by *Spirochaetaceae* [41, 42]. An early 16S rRNA gene clone library study on two species of Mediterranean sea pens (*Pennatula phosphorea* and *Pteroeides spinosum*) showed the dominance of *Alphaproteobacteria*, *Gammaproteobacteria,* and *Mollicutes*, with distinct communities in mucus and different tissue compartments at the phylum level [43]. A more recent study comparing bacterial communities across the two octocoral orders in the NE Atlantic found that *Endozoicomonadaceae* dominated the microbiomes of Malacalcyonacea (~76% mean relative abundance), whereas Scleralcyonacea (e.g. *Callogorgia*, *Ellisella*, *Isidella*, *Funiculina*) harbored more diverse and heterogeneous bacterial communities dominated by different families, including *Flavobacteriaceae*, *Spongiibacteraceae*, *Rhodobacteraceae*, and *Mycoplasmataceae*, depending on host species. These communities were also shaped by environmental context, including depth and nutrient availability [44].

Several studies reported seasonal and geographical stability among octocoral-associated bacterial communities [30, 34, 41, 42, 45]. Temporal analysis of bacterial community dynamics of a dozen temperate [30, 42], subtropical and tropical species [30, 34, 45] showed overall stable bacterial associations over time, while compositional changes primarily involved rare bacteria. A multi-year dataset from four Florida octocoral species revealed that host species identity drove microbiome composition, with temporal variation explaining only 1.8% of the total variation, and core symbionts (*Endozoicomonadaceae*) being stable over years [45]*.* In Mediterranean gorgonians, more pronounced geographical rather than temporal variation in bacterial community composition has been observed, potentially suggesting bacterial adjustment to local environmental conditions [41, 42]. However, emerging evidence suggests that host-specific traits can modulate temporal dynamics, warranting caution in generalizing patterns of microbiome stability. Comparison among multiple Australian soft coral species showed that some exhibited seasonal shifts in bacterial community composition, whereas others maintained stable bacteriomes [30]. While bacterial diversity remained relatively constant across seasons and years in shallow-water Caribbean gorgonians, relative abundances of dominant bacterial taxa fluctuated significantly over time [34]. Therefore, more long-term baseline data spanning large geographic areas are needed to fully understand microbiome stability across time and space.

Currently available genomes of octocoral-associated bacteria span eight classes and >20 families (Fig. 2), consistent with taxa commonly reported in both cultivation-dependent and -independent studies. Thus, the genomes capture substantial diversity, although key groups such as the *Spirochaetaceae* remain absent. Most bacterial genomes were obtained from whole octocoral fragments or tissue macerates, and although several studies report removal of epibionts, this offers limited resolution of the exact microhabitat of bacteria within octocorals (Table S1). Genome sizes vary markedly, from highly reduced genomes <0.8 Mb in intracellular *Mycoplasmatales* symbionts to >7 Mb in *Roseibium album* strains (Fig. 2), indicating pronounced genome expansion and reduction dynamics that likely reflect obligate versus facultative symbiotic lifestyles. We found a striking balance between isolate genomes (*N* = 39; 49%) and metagenome-assembled genomes (MAGs)/single-amplified genomes (SAGs) (*N* = 40; 51%). This highlights an equitable effort by the scientific community to understanding the octocoral bacteriome and its functional potential using both cultivation-dependent and cultivation-independent approaches, enabling a more comprehensive assessment. MAG-based approaches typically target the dominant, yet often hard-to-cultivate, associates, delivering insights into putative symbiotic functions. MAGs from low-abundance taxa are usually underrepresented due to sequencing depth and coverage limitations. Contrarily, cultivation-based approaches tend to favor the less abundant phylotypes, offering complementary insights into metabolic versatility [e.g. 46]. Access to cultured representatives facilitates microbiome manipulation studies for experimental validation of functions on the strain level. The available genomic data are, however, strongly biased toward the Atlantic Ocean, temperate gorgonians, and healthy hosts (Fig. 2). In contrast, genomes representing the pathobiome, tropical and deep-sea habitats, and other octocoral lineages remain scarce. No bacterial genome records are currently available for octocorals of the order Scleralcyonacea, nor from samples collected in the Indian, Southern, or Arctic Oceans, or the Mediterranean and Red Sea—regions that are critical for understanding coral holobiont responses to climate change. Moreover, the currently available bacterial genomes are derived from only ca. 11 host species (Fig. 2), in stark contrast to the >3000 known extant octocoral species [3], leaving major gaps in our understanding of functional conservation across hosts and ecosystems.

**Figure 2:**
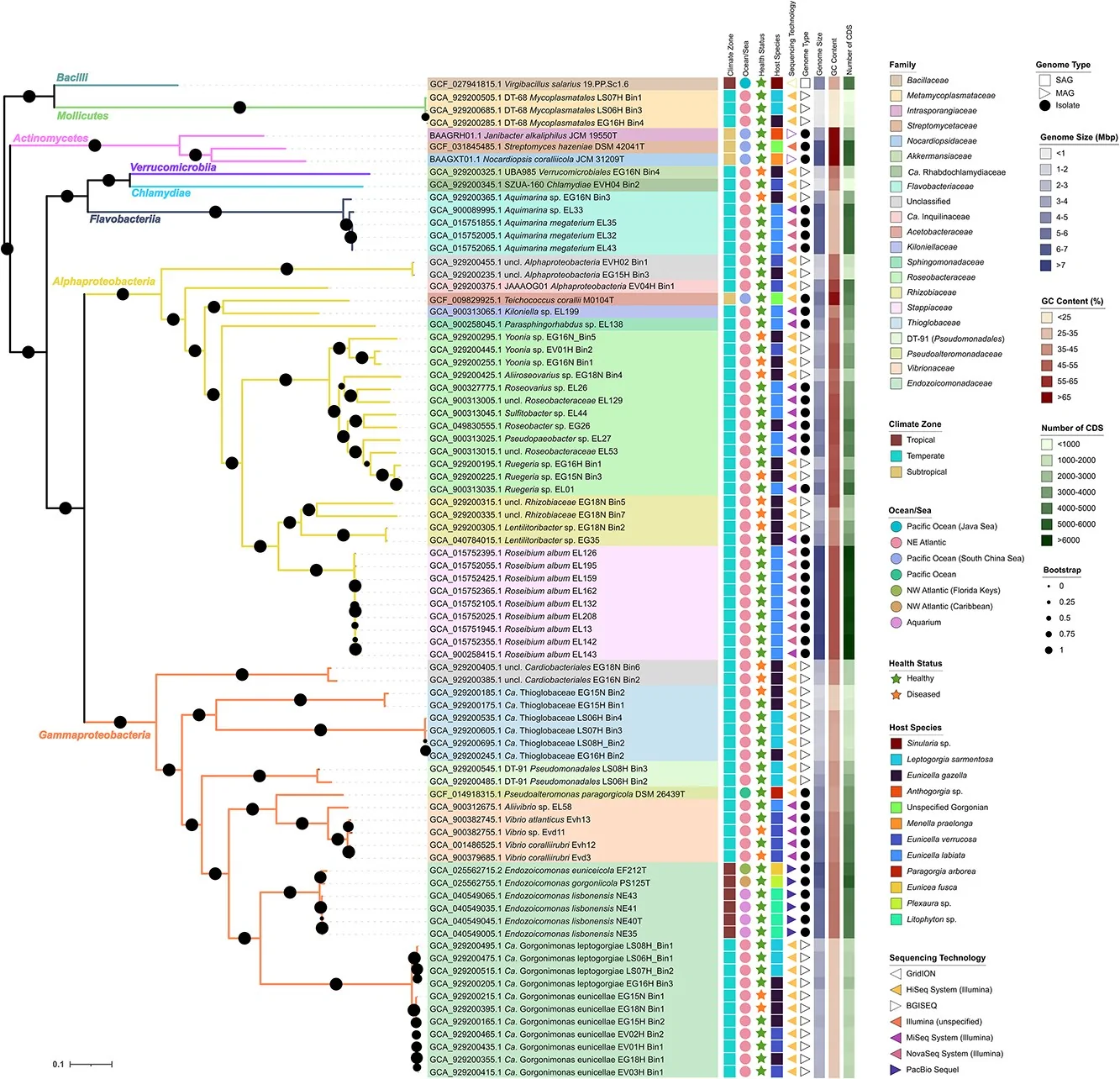
Phylogenomic relationships, genome characteristics, provenance, and other metadata of publicly available genomes from octocoral associated bacteria. All 79 genomes, MAGs, and SAGs shown here are available in NCBI (last accessed 8 September 2025) and meet the following quality criteria: >60% genome completeness, <5% contamination (assessed by CheckM1) and an overall quality score (%Completeness–5 × %Contamination) above 50%. The phylogenomic tree was constructed in the DOE Systems Biology Knowledgebase (KBase) platform using SpeciesTreeBuilder v.0.1.4 (“Insert Set of Genomes into Species Tree”), after annotation of the genomes with Prokka. The alignment was based on 49 core clusters of orthologous groups (COGs) of proteins. Tree inference was performed using FastTree2 with the maximum-likelihood method and the Jukes–Cantor evolutionary model with CAT approximation, including 1000 bootstrap replicates. Filled circles on branches indicate bootstrap support ≥70%. Tree branches and genomes are color-coded by class and family-level taxonomy, respectively, with genome accession numbers listed prior to genome names. Genome metadata, i.e. geographic origin, host health status, host species, sequencing technology, genome type, genome size, GC content, and coding sequence (CDS) counts are shown in columns adjacent to the tree. Styling of the phylogenomic tree was done in iTOL v.7 and Inkscape. Detailed methodology on the phylogenomic inference can be found in Additional File 1. Genome quality metrics, genomic features, and full metadata are provided in Additional Table S1.

The diversity and abundance of archaea in octocorals appears to be much lower than those of bacteria. Octocoral-associated archaea have received little attention and thus, their roles and contributions in the octocoral holobiont remain poorly understood. In the Atlantic gorgonian *Eunicella labiata*, archaeal populations contributed only about 0.6% relative abundance of the prokaryotic community, with most phylotypes belonging to the order *Thermoplasmatales* [46]. The phylum *Thermoproteota* was also observed and mostly represented by the ammonium oxidizing *Nitrosopumilus*, suggesting a role of archaea in nitrification in some octocorals [46]. Congruently, O’Brian *et al.* [47] reported *Nitrosopumilaceae* from octocorals at the Great Barrier Reef. Although no archaeal genomes or MAGs are yet available from octocorals, multiple *Thermoproteota* MAGs carrying ammonia monooxygenase (*amoA*) genes, central to ammonia oxidation, have been recovered from scleractinian corals, reinforcing a putative role in nitrogen cycling [48, 49].

#### Microeukaryotes

Many shallow water octocorals establish mutualistic relationships with photosynthetic dinoflagellates from the Symbiodiniaceae family (Fig. 1a), mostly with *Symbiodinium*, *Breviolum*, *Cladocopium*, *Durusdinium*, *Philozoon,* and *Gerakladium* [13, 27, 28, 50]. In general, the diversity of Symbiodiniaceae associated with octocorals is higher in the tropics compared to temperate regions [27, 28]. The octocorals studied to date frequently host only one dominant algal genus, with these dominant Symbiodiniaceae genera exhibiting distinct geographical clustering patterns and strong host specificity [27, 28].

Beyond the Symbiodiniaceae, microeukaryotes (i.e. the eukaryome) have been poorly investigated. This challenge primarily stems from the difficulty of culturing microeukaryotes, and the technical constraints associated with ITS1/2 region and 18S rRNA gene-based sequencing, such as cross-amplification of host DNA. As a result, protocol refinements such as blocking primers interfering with host 18S rRNA gene amplification are typically required to characterize the microeukaryotic community of octocoral [51, 52]. Despite these technical challenges, recent DNA-based studies have highlighted the significant diversity of the octocoral eukaryome, which can include up to thousands of different ASVs in the case of the Mediterranean gorgonians *C. rubrum* and *Paramuricea clavata* [51–53]. Dominant microeukaryotes of both species included Syndiniales Dino-Group I dinoflagellates, licnophorid ciliates, and corallicolid apicomplexans. Certain Apicomplexa-like lineages have a nearly exclusive association with coral species, including the octocoral *Paramuricea placomus* [54]. Corallicolids, a group of obligate intracellular parasitic *Apicomplexa*, were observed in cold-water octocorals, specifically in mesophotic and deep-sea taxa such as *Acanthogorgia aspera*, *Callogorgia delta*, *Ellisella* sp., *Muricea pendula, Paramuricea* sp., *Paragorgia* sp., *Swiftia exserta*, and unidentified bamboo octocorals (Isididae) [55]. While the mechanisms of interactions between corallicolids and octocorals remain unknown, they are among the most abundant non-photosynthetic microeukaryotes in scleractinians where their biology is slightly better understood [56]. Transcriptomic responses of *Acropora digitifera* larvae to *Chromera velia* resemble pathogen or parasite infections rather than mutualistic responses to *Symbiodinium*, suggesting that coral-associated apicomplexan relatives are unlikely to be beneficial [57].

Even with reports of devastating opportunistic infections with the fungus *Aspergillus*, known as sea fan aspergillosis, in Caribbean and Eastern Pacific gorgonians (Fig. 1b) [58–60], there are few high-throughput gene sequencing studies on fungal communities in healthy octocorals. Most fungi-related work is culture-dependent and focused on their secondary metabolites and bioactivities of pharmaceutical and biotechnological value (reviewed in [20–22]). Frequently isolated fungi belong to the main divisions of Ascomycota and Basidiomycota, specifically members of *Aspergillus* and *Penicillium* from octocorals around the globe [22]. Less frequently, members of the genera *Cladosporium*, *Tritirachium*, *Nigrospora*, and *Fusarium* are reported [20, 22, 60–62]. In general, local environmental conditions seem to be a main driver of fungal community composition in healthy and *Aspergillus*-infected octocorals [61, 62], with sea fan aspergillosis outbreaks peaking in the aftermath of marine heat waves [63].

#### Viruses

Research on cnidarian-associated viral communities, or viromes, is scarce. Most viruses associated with octocorals are bacteriophages of heterotrophic bacteria and cyanobacteria, followed by viruses belonging to the families Phycodnaviridae [64], Parvoviridae, Orthototiviridae, and Circoviridae [65]. Dicistroviridae, a family of small RNA viruses, was reported in *Xenia* sp. [66]. Phycodnaviridae are known to infect algae and were previously found in both bleached and visibly healthy scleractinian corals [67]. While specific interactions between this family of viruses with other members of the octocoral holobiont remain to be determined, their increased relative abundances in bleached corals suggests a potential role in, or response to the loss of Symbiodiniaceae under stress. Higher relative abundances and changes in composition of viral contigs were reported in diseased compared to healthy *Gorgonia ventalina* [65], indicating that octocoral-associated viromes are correlated with host health. Moreover, microbial metagenomes of healthy *Eunicella gazella* are characterized by higher abundances of restriction-endonucleases and CRISPR/Cas proteins that function as effective anti-phage defense systems (Fig. 1a). This may indicate that the “Piggyback-the-Winner” model governs phage–bacteria interactions within the octocoral holobiont, as has been proposed also for scleractinians and other hosts [68]. This model supports the prevalence of lysogenic phage cycles within coral microbiomes in comparison with seawater microbiomes, where a lytic mode of phage–bacteria interaction prevails.

An ancestor of *Mimivirus*, a giant DNA virus, has also been found to infect octocorals [69]. The mitochondrial gene in octocorals, *mtMutS,* potentially associated with DNA mismatch repair, was previously proposed to likely originate from horizontal gene transfer from either *Epsilonproteobacteria* or a large DNA virus. This highlights the possibility of to date unknown mechanisms by which metazoan mitochondrial genomes may evolve [70]. Still, almost nothing is known about the actual functions, organismal interactions, or ecological relevance of viral communities in octocorals, a prime avenue for future research. More advanced research on scleractinians can serve as inspiration. Controlled microcosm experiments have demonstrated that bacteriophage addition reshapes microbial community composition, enhances diversity, and influences elemental cycling within the scleractinian holobiont [71]. Future viral manipulation experiments such as phage reduction or enrichment assays could test whether bacteriophages structure dominant bacterial populations and modulate nutrient cycling in octocorals.

### Localization and spatial partitioning of octocoral-associated microorganisms

Microbial communities exhibit spatial partitioning across distinct microenvironments within the octocoral holobiont, including the surface mucus layer, gastrodermis, mesoglea, and gastrovascular cavity [72]. Each compartment has distinct chemical gradients of oxygen, pH, and redox potential, alongside differential nutrient availability, and varying degrees of exchange with the surrounding seawater [73] that shape symbiont-driven processes like nitrogen fixation or sulfur cycling. The spatial localization of microbial associates within the host is therefore a fundamental conceptual framework that is critical for understanding symbiont functions, interactions, and lifestyles. The mucus typically harbors a more variable, seawater-influenced bacterial community, whereas tissue-associated microbiomes tend to be more stable and host-specific [72]. In symbiotic octocorals, Symbiodiniaceae reside intracellularly in host-derived symbiosomes within specialized gastrodermal cells in the polyps and the coenenchyme. Although symbiosome regulation has been studied primarily in scleractinian corals and anemones, these modified phagosomes are considered a conserved feature of cnidarian–dinoflagellate symbioses, preventing symbiont digestion while providing a regulated interface for nutrient exchange, immune modulation, and symbiotic maintenance [74]. Histological studies indicate that octocorals share this fundamental cellular organization, suggesting similar underlying mechanisms [75]. However, despite increasing recognition of the importance of spatial organization, most octocoral microbiome studies rely on bulk tissue sampling, which obscures fine-scale spatial patterns. High-resolution imaging techniques, such as fluorescence *in situ* hybridization (FISH), compartment-specific sequencing, isotopic tracing, and micro-sensing, offer promising approaches but remain underutilized in octocorals. As a result, direct evidence linking specific microbial taxa to defined host microenvironments remains limited. This is largely due to technical challenges, including low sample biomass, microscale resolution constraints, high autofluorescence, and non-specific binding of FISH probes, which make such analyses labor-intensive and costly [76].

Nevertheless, a few studies have provided initial insights into the localization of octocoral-associated microorganisms. Similar to observations in scleractinian corals [77], La Rivière *et al.* [78] identified aggregates of tightly packed Gram-negative cells (~1 μm in diameter) within the endodermis of polyps of the gorgonian *P. clavata* using universal eubacterial FISH probes. These coral-associated microbial aggregates (CAMAs), measuring 12–20 μm in length, were hypothesized to belong to *Endozoicomonas*, a dominant genus in *P. clavata* [78]. Supporting this interpretation, FISH–CLSM studies in scleractinians and other marine invertebrates have demonstrated that *Endozoicomonadaceae* form dense aggregates within host tissues (reviewed in [79]). Moreover, in the red coral *C. rubrum*, *Endozoicomonas* phylotypes were also primarily localized in the polyps [80], positioning them close to sites of heterotrophic ingestion and implicating them in holobiont nutrient cycling.

Several bacterial MAGs from temperate gorgonians encode metabolic pathways associated with anaerobic respiration and pyruvate fermentation to lactate, processes that occur under anaerobic conditions [23]. This suggests the presence of suboxic niches within the octocoral holobiont, as proposed for other cnidarians [81]. “GenomeSPOT” [82] analysis of the 79 octocoral-associated bacterial genomes presented here predicted low oxygen tolerance for all *Ca*. Gorgonimonas spp. (*Endozoicomonadaceae*), *Endozoicomonas euniceicola*, one *Chlamydiae*-, and one *Mycoplasmatales* phylotype, suggesting preference for microaerophilic or anaerobic microhabitats (Fig. 3). Finally, recent work on the deep-sea octocoral *Callogorgia delta* demonstrated that dominant *Mollicutes* symbionts reside within the mesoglea of polyps [83]. The pronounced genome reduction and metabolic dependency observed in these *Mollicutes* indicate long-term adaptation to protected, host-associated niches. Future research will benefit from leveraging (meta)genomic resources to design clade-, phylotype-, or functional gene-specific (CARD-)FISH probes. This will enable systematic identification of bacterial symbionts, their sites of association, and aggregation behavior. The genomes presented here provide an excellent starting point, particularly those from dominant microbiome members (Table S1), including *Ca.* Gorgonimonas spp., *Mycoplasmatales*, *Ruegeria* symbionts of temperate gorgonians [23], and *Endozoicomonas lisbonensis* from *Litophyton* sp. [31]. A tissue- and cell-resolved perspective will help decipher the niche-specific interactions that drive holobiont physiology, moving past global microbiome profiles to uncover the mechanistic basis of complex octocoral–microbial associations.

**Figure 3:**
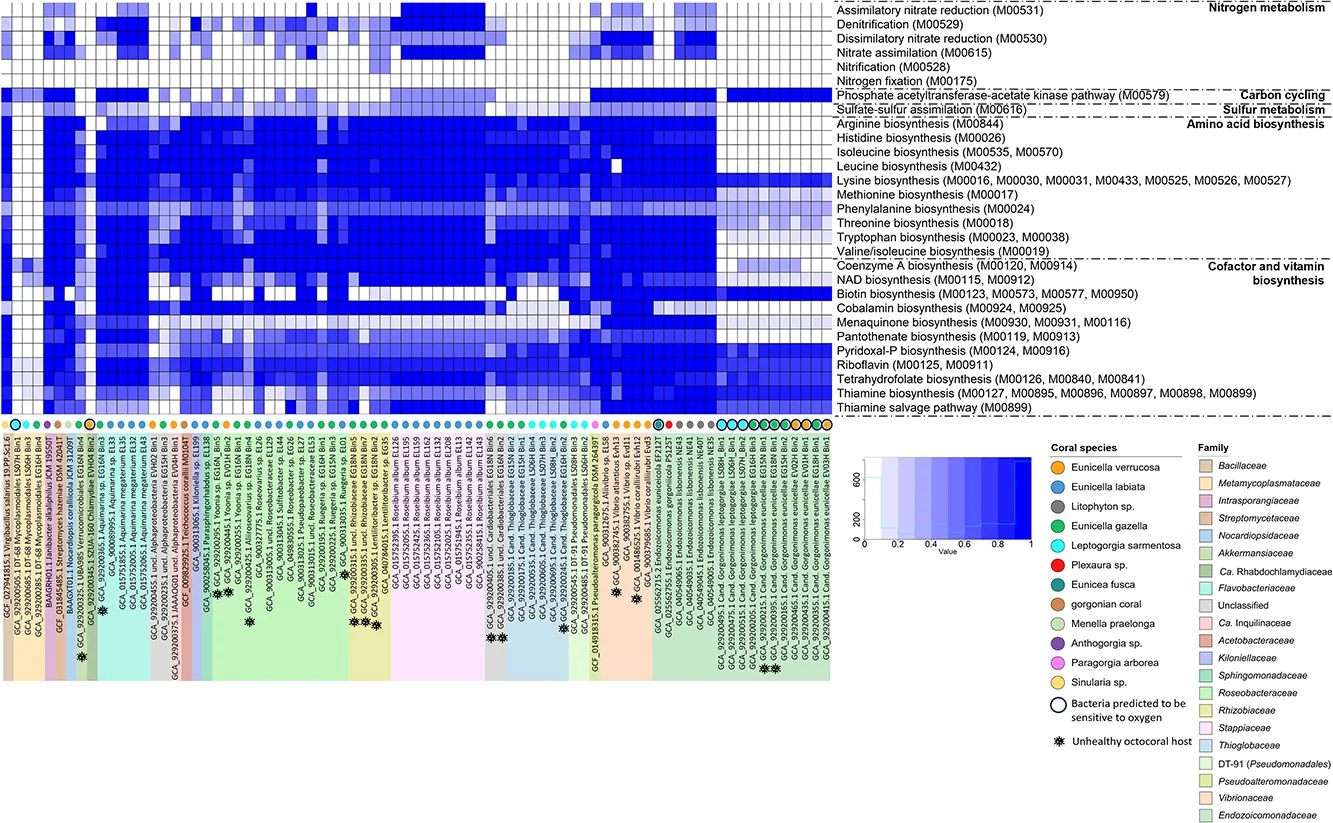
Heatmap showing completeness of KEGG metabolic modules associated with nitrogen, carbon, and sulfur cycling, as well as amino acid, vitamin, and cofactor biosynthesis in 79 genomes of octocoral-associated bacteria. Module completeness (0–1) was estimated in the program Anvi’o v.8 from gene presence/absence, where dark blue indicates complete or near-complete modules and white absent modules. Genomes are ordered and colored according to their phylogenomic relationships (see Fig. 2). The octocoral host from which the bacteria were derived is indicated by colored circles, while black rings surrounding these circles denote bacteria predicted to be oxygen-sensitive (GenomeSPOT analysis). For each bacterial genome/MAG, contig databases were generated and annotated using *anvi-gen-contigs-database*, *anvi-run-hmms*, and *anvi-run-kegg-kofams* with default parameters. Module completeness was derived using *anvi-estimate-metabolism*. Only selected KEGG modules within nitrogen, carbon, and sulfur metabolism, amino acid, and cofactor/vitamin metabolism were retained; modules representing the same biosynthetic pathway were grouped, and the highest completeness value within each group is shown. The heatmap was produced in R (v.4.5.1) using the *reshape2* and *gplots* (heatmap.2) packages. See Additional File 1 for detailed methodology on KEGG annotation and Additional Table S3 for KEGG profiles.

### Functional roles of the octocoral microbiome

While high-throughput ribosomal gene sequencing has revealed key insights into microbial community composition and dynamics, it provides limited information on microbial functions, interactions, and lifestyles. Functional approaches are therefore essential to elucidate microbial ecology in octocoral holobionts. The above discussed diversity of microbial groups likely underpins dynamic cross-domain metabolic interactions among prokaryotes, microeukaryotes, viruses, Symbiodiniaceae, and the octocoral host. The nature and mechanisms of these interactions, and the metabolic properties they generate, remain largely unresolved. Based on other host–microbe systems, such interactions are likely tightly regulated, highly complex, and adaptive, supporting microbiome stability, pathogen resistance, and stress tolerance under environmental change [84], potentially contributing to the greater resilience of several octocoral species relative to scleractinians.

#### Carbon supply and cycling

In the tropics and subtropics, Symbiodiniaceae symbiosis is common, while most temperate species (~90% in the Mediterranean and North Atlantic) are exclusively heterotrophic [85]. Consequently, Symbiodiniaceae communities remain largely uncharacterized in temperate octocorals, likely reflecting the high proportion of non-photosymbiotic members among the commonly studied species. Symbiodiniaceae can supply octocorals with a substantial portion of photosynthates. Host morphology affects autotrophic carbon acquisition, with thin-branched, small-polyp species being more autotrophic than massive, large-polyp forms [17, 86]. Photosynthate translocation rates vary widely among octocoral species, Symbiodiniaceae genera, and habitats, ranging from 10% in *Capnella gabonensis* to 75% in *Sinularia flexibilis* and 85% in *Rhytisma fulvum fulvum* [15, 87]. Autotrophic carbon uptake in octocorals does not decrease markedly with depth, owing to photoacclimation mechanisms whereby Symbiodiniaceae adjust their chlorophyll content, symbiont density, and light-use efficiency [15, 88, 89]. Moreover, their lower optimum irradiance compared to scleractinians confers an advantage in deeper environments [90]. The overall contribution of photosynthetically fixed carbon to the energy requirements of octocorals remains poorly understood. Octocorals are generally less autotrophic than scleractinians, likely due to a lower surface area-to-volume ratio, affecting gas exchange and light exposure [91]. This applies to both carbon and inorganic nitrogen fluxes: ammonium and nitrate uptake rates per ash-free dry weight are at least an order of magnitude lower in octocorals [92]. Consequently, octocorals may rely more on heterotrophic feeding, with dependence varying by species, health, depth, and season [91].

Particulate organic matter (POM) ingestion can meet on average 160% of the metabolic requirements of non-photosymbiotic octocorals, versus ~30% in photosymbiotic holobionts [93]. Octocorals feed on a broad spectrum of organic matter, including dissolved organic matter (DOM) or detrital POM, bacterioplankton, and larger planktonic organisms such as diatoms, dinoflagellates, or zooplankton, whereby feeding preferences are species- and context-dependent [94, 95]. Remarkable poly-carbohydrate cycling capabilities were observed in several bacterial symbionts of octocorals [23]. All *Ca.* Gorgonimonas (*Endozoicomonadaceae*) MAGs of temperate gorgonians encode chitinases and chitin-binding proteins, indicating that these symbionts can hydrolyse chitin, the most abundant marine polysaccharide [23, 96]. Chitinase genes and chitin-degrading ability occur also in *Endozoicomonas* isolates of tropical *Litophyton* sp. [97, 98], indicating conserved functions across octocoral holobionts. Chitinase genes are generally widespread among *Endozoicomonadaceae*, suggesting a central role in coral chitin processing [96]. Of the 79 octocoral-associated bacterial genomes presented here, over 90% derived from non-photosymbiotic hosts (Table S1) and 51% encode chitinase genes (Table S2). Given that chitin is abundant in the natural zooplankton and phytoplankton diet of octocorals, and both a carbon and nitrogen source, a microbiota specialized in chitin degradation may provide a nutritional advantage to heterotrophic octocorals or under carbon-limited conditions. Chitinases may also protect against chitin-containing fungal pathogens [13], or mediate host–bacteria signaling during colonization [99].

#### Cycling of nitrogen, sulfur, and other nutrients

Nitrogen cycling, a key process in scleractinians, is less studied in octocorals. Symbiodiniaceae associated with octocorals can use multiple inorganic and organic nitrogen sources [92]. As in other cnidarian–dinoflagellate symbioses, ammonium is preferred, as it can be directly incorporated into amino acids, while nitrate assimilation is slower due to the required reduction steps. Organic nitrogen uptake has been observed but tends to be more variable and may involve greater host mediation. Symbiodiniaceae translocate nitrogen-rich compounds, such as amino acids, to the host, contributing to growth and metabolic balance. However, octocorals generally rely more on heterotrophy than many scleractinians, resulting in nitrogen uptake rates an order of magnitude lower [92]. Octocorals also exhibit some of the lowest diazotrophy (nitrogen fixation) rates reported on reefs [100–103], making only negligible contributions to biological nitrogen fixation at reef-scale, across reef habitats, and seasons [101, 103]. These differences are likely due to the porous calcareous skeleton of scleractinians, which hosts endolithic nitrogen-fixing prokaryotes, a feature absent in soft corals, which lack a true calcareous skeleton and endoliths [103]. In contrast, octocorals show among the highest denitrification rates among several benthic organisms and substrates on reefs, likely facilitated by a thicker surface mucus layer in certain octocorals (e.g. *Xenia* spp., *Litophyton* spp.) compared to scleractinians, which may create localized low-oxygen microenvironments that support denitrification [100]. These physiological patterns are reflected in microbial functional gene repertoires. Denitrification genes are widespread across available octocoral-associated bacterial genomes and MAGs, including *R. album*, *Aquimarina megaterium*, several *Roseobacteraceae* (e.g. *Ruegeria* [104]), and *Kiloniella* spp. (Fig. 3). Other nitrogen cycling pathways, including nitrate assimilation, are also represented, while our meta-analysis did not identify any nitrogen fixation-related genes (Fig. 3). Moreover, many *Endozoicomonadaceae* have been experimentally shown to possess nitrate reduction capabilities [79, 98]. Together, this suggests fundamental differences in holobiont nitrogen transformations in octocorals compared to scleractinians [105]. Denitrification may play a substantial and potentially advantageous role in octocoral holobiont functioning for example by preventing excessive nitrogen accumulation and helping maintain nutrient balance under conditions of elevated nutrient input or eutrophication. However, effects likely vary across species and environmental conditions. More comparative studies linking microbial functional gene content and biogeochemical rate measurements with host physiology, are required to fully comprehend these processes, especially in the context of environmental change. For example, in the mixotrophic soft coral *Xenia umbellata*, heat and elevated dissolved organic carbon increased the relative abundance of diazotrophs. In contrast, the more autotrophic *Pinnigorgia flava* showed a weaker response that occurs only under combined stress conditions, rather than under heat stress alone [24].

Octocoral-associated microbiomes are also likely to contribute to sulfur cycling. Incomplete sulfate and sulfur assimilation pathways occur widely among *Endozoicomonadaceae*, *Vibrionaceae*, *Ca.* Thioglobaceae, *Roseobacteraceae*, and *Aquimarina* [23] (Fig. 3). Taurine utilization pathways and its metabolization to aminoacetaldehyde and sulfite, via taurine deoxygenase (TauD), or to sulfoacetaldehyde via taurine–pyruvate–aminotransferase are also reported among cultured and uncultured octocoral symbionts [23, 106, 107]. Taurine is an amino sulfonic acid commonly found in animal tissues and produced by corals, where it also plays a role in controlling photosynthesis and the release of carbon fixation products by Symbiodiniaceae [108]. Taurine-degrading bacteria may have a regulatory influence on this process, highlighting the complexity of interaction mechanisms between various members of the microbiome and their host.

Microbiomes of the deep-sea octocorals *A. aspera* and *Acanthogorgia spissa* are enriched in genes related to mercury detoxification, ammonia oxidation, organophosphorus degradation, and degradation of polycyclic aromatics [109]. Aromatic compound and halogen degradation pathways are prevalent in deep-sea habitats due to the accumulation of halogenated organic compounds through the biological carbon pump [110]. Genes involved in the degradation of polycyclic aromatic hydrocarbons and phenolic compounds were detected in the genomes of *Sphingorhabdus* and *Roseobacter* strains associated with temperate *Eunicella* spp., respectively, suggesting potentially beneficial roles in environmental detoxification and pollutant removal [106, 111].

#### Amino acid, vitamin, and micronutrient supply

The microbiome of healthy gorgonians (*Eunicella* spp. and *Leptogorgia sarmentosa*) is enriched in iron-storage proteins, ferritin and bacterioferritin, and genes for magnesium and cobalt transporters [29]. These metals are essential cofactors for numerous enzymes and organic molecules. Cobalt is a key component of vitamin B12 (cobalamin), which is essential for both the coral host and the Symbiodiniaceae [112], but can only be synthesized by prokaryotes. Octocoral-associated bacterial genomes and MAGs show biosynthesis capacity for many essential amino acids (arginine, histidine, valine/isoleucine, leucine, lysine, methionine, phenylalanine, threonine, and tryptophan), cofactors (coenzyme A, NAD), and vitamins (B1, B2, B5, B6, B9, B12, K) (Fig. 3). Metabolic functions are typically partitioned across distinct bacterial taxa rather than all occurring in a single organism, suggesting metabolic specialization and niche partitioning within the holobiont [23]. This may enable genome streamlining in host-adapted lineages, such as *Ca.* Gorgonimonas and *Mycoplasmatales*, which show reduced genome content (Fig. 2), and lack several amino acid- and vitamin biosynthesis pathways (Fig. 3) [23]. Such metabolic deficiencies likely contribute to their current uncultivability and imply obligate dependence on metabolic cross-feeding within the octocoral holobiont. Further supporting a role of octocoral-associated bacteria in micronutrient provisioning, *Endozoicomonas* strains isolated from octocorals have demonstrated a high capacity to chelate environmental iron in laboratory assays [98].

#### Antimicrobial compound production and defense

Recent studies demonstrate diverse associations between octocorals and marine fungi. While some fungal taxa act as opportunistic pathogens linked to sea fan aspergillosis [58, 59] (Fig. 1), others could benefit the holobiont through chemical defense, owing to their immense chemodiversity [20–22, 113]. Metabolites from octocoral-derived *Aspergillus* strains display cytotoxic, antioxidant, antimicrobial, and antiviral activities [20, 21]. The production of bioactive compounds sydowinol, sydowinin A and B, and sydowic acid has been linked to pathogenicity in *Aspergillus sydowii* strains from octocorals [114]. Octocoral-associated fungi produce sesqui-, di- and triterpenoids, alkaloids, steroids, linear peptides, and aromatic polyketides [reviewed in 20, 21, 22]. Members of *Penicillium* may additionally supply gorgonians with several hydroxylated fatty acids [115]. Although fungi-derived fatty acids have key roles in cell membrane structure, energy storage, and host fitness in reef-building corals [116], their specific functions and energetic contributions to coral holobiont health remain unclear. Overall, the breadth of bioactivity and chemodiversity suggests that octocoral-associated fungi may influence microbial community structure, and thus host functioning [discussed in detail in 22, 114]. Microbiome manipulations testing the effects of fungi and/or their metabolites on host health under thermal stress or pathogen challenge could help elucidate fungal roles in octocorals. To disentangle interactions between fungi and other microbiome members, such as bacteria, reciprocal suppression experiments could be used to assess whether these relationships are competitive or collaborative.

Bacterial symbionts likely contribute further to host chemical defense, opportunist control and/or microbiome regulation. Around 60 novel secondary metabolites are reported each year from octocoral-associated microorganisms [20]. Although ascomycete fungi clearly dominate, *Actinomycetota* (*Streptomyces* spp.) and *Bacillota* (*Bacillus* spp.) bacteria are also among the producers [20]*.* Genome analyses of *Eunicella*-associated *Aquimarina*, *Vibrio*, *Roseibium*, *Ruegeria,* and other *Alphaproteobacteria* revealed diverse secondary metabolite biosynthetic gene clusters (BGCs) (Fig. 4), including polyketide synthases (PKS), non-ribosomal peptide synthetases (NRPS), betalactones and thiopeptides, all commonly linked to inhibitory activities [21, 107, 117]. Gorgonian-derived MAGs of an uncultured *Alphaproteobacteria* lineage (*Ca.* Inquilinaceae) were enriched in PKS, bacteriocin, and NRPS BGCs, one matching the antibacterial cyclic depsipeptide icosalide (100% similarity) [23]. Likewise, unclassified *Pseudomonadales* (DT-91 clade) MAGs from *L. sarmentosa* carried multiple NRPS BGCs, one with 100% similarity to the antimicrobial bicornutin A [23]. Certain bacterial symbionts may therefore specialize in antimicrobial compound production, whereas others such as *Endozoicomonadaceae* and *Mycoplasmatales,* which have a more intracellular lifestyle*,* may have lost this capacity and instead fulfil roles related to nutrient cycling [23]. Meta-analysis of the 79 octocoral-associated bacterial genomes analysed in this study confirmed substantial variation in BGC counts, ranging from zero to 30 BGCs (Fig. 4), with the highest numbers detected in gorgonian-derived *Streptomyces hazeniae* from the South China Sea, a taxon and region well known for its high natural product diversity [20]. RiPP-like BGCs were highly prevalent among the 79 genomes. This class includes bacteriocins, which are commonly involved in bacterial competition and may contribute to structuring the octocoral bacteriome or preventing the establishment of invading pathogens. *Eunicella*-associated *Aquimarina* spp. inhibit conspecific *Vibrio* spp. strains in co-culture assays [117], suggesting a role in opportunist control. A *Pseudoalteromonas* strain from healthy *Leptogorgia alba* produces the polyketide alteramide A, which shows light-dependent antifungal activity [25]. In darkness, both *Pseudoalteromonas* and pure alteramide A inhibit *Penicillium citrinum* isolated from necrotic *Eunicea* sp. tissue and other fungi. More alteramide A is produced in the dark, while light exposure inactivates it via photo-induced cyclization, potentially protecting the host from infections during nocturnal heterotrophic feeding, when polyps are more exposed [25].

**Figure 4:**
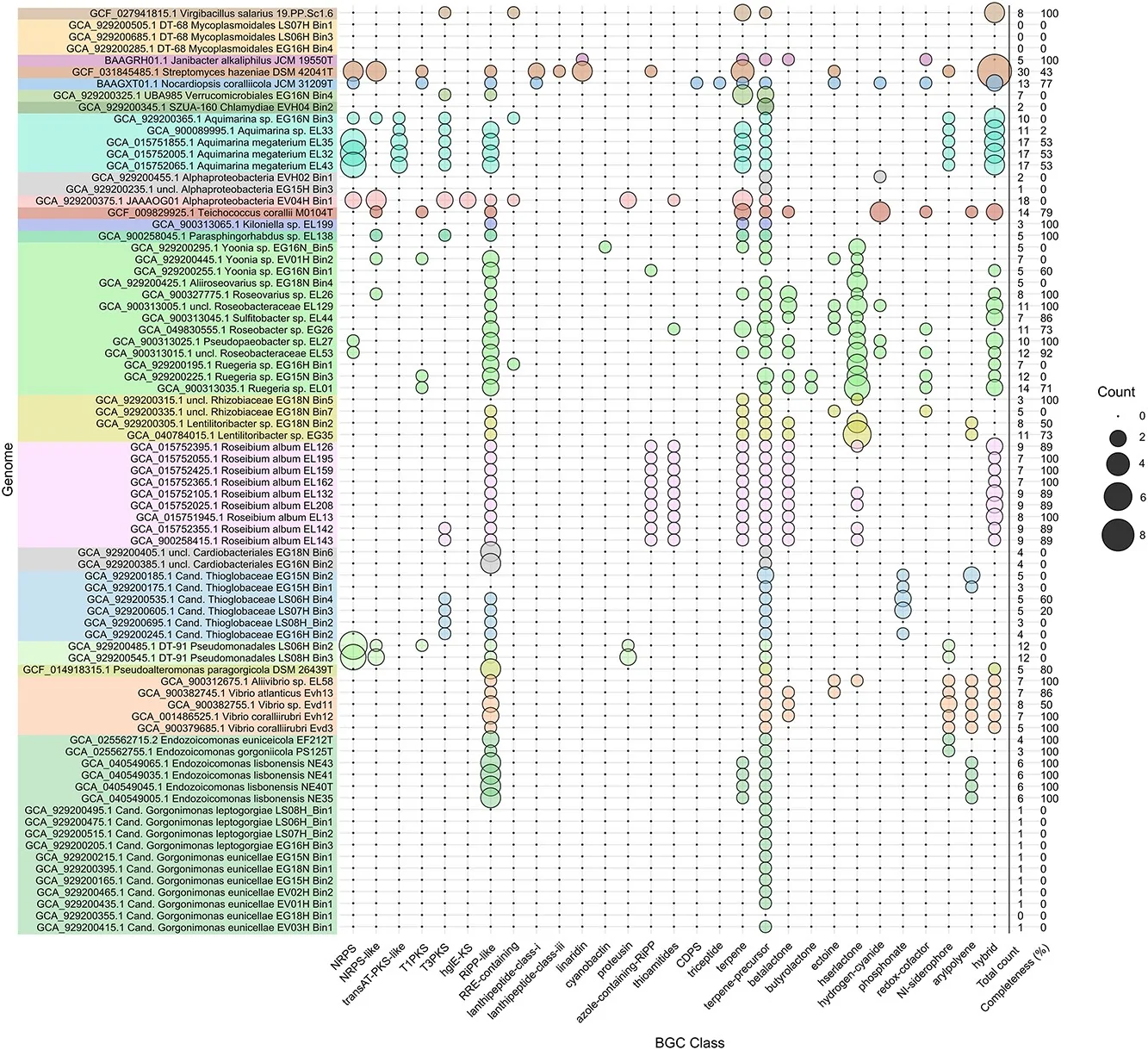
BGCs encoding secondary metabolites in 79 genomes of octocoral-associated bacteria. BGCs were annotated using antiSMASH v.8.0.4 [118] via the multiSMASH wrapper (10.5281/zenodo.8276143) with default parameters. The total number of BGCs and the proportion predicted to be complete (i.e. not located at contig edges) are shown in the column on the right. NRPS—nonribosomal peptide synthetase; PKS—polyketide synthase; RiPP—ribosomally synthesized and post-translationally modified peptides; PRE-containing—promotor recognition element-containing BGC (for RiPP biosynthesis); CDPS—cyclodipeptide synthase. Hybrid clusters are defined as those containing features from more than one compound class. The full BGC profile for each genome is provided in Additional Table S4.

#### Host colonization and symbiosis establishment

Full metagenome and genome surveys have provided insights into the likely roles and mechanisms of host–bacteria interactions in octocorals [23, 29, 97, 107]. Microbial metagenomes of temperate gorgonians show high abundances of genes related to eukaryotic-like proteins (ELPs, especially ankyrin and WD40 repeats) and type III secretion systems, likely involved in host–symbiont recognition, host colonization, and symbiosis establishment [29]. Likewise, gorgonian-derived MAGs of yet uncultured *Endozoicomonadaceae* (*Ca.* Gorgonimonas) symbionts are enriched in ankyrin repeats, type III secretion systems, serine/threonine protein kinases, serine protease inhibitors, YejM, and type IV pili [23]. Genomes of cultured octocoral-associated *Endozoicomonas*, *Roseobacter*, and *Roseibium* also encode ELPs and diverse secretion systems typical of host-associated lifestyles [97, 106, 107].

Bacteria associated with octocorals may be transmitted vertically, from parent to larva, and/or horizontally, from the environment [119, 120]. Vertical transmission allows early colonization with potentially important symbionts that may support larval development, while horizontal acquisition allows for local adaptation. Indeed, ambient reef conditions shaped bacterial communities in blue coral (*Heliopora coerulea*) larvae, but not maternal colonies with established microbiomes [121]. While larvae inherit portions of the parental microbiome in *R. fulvum fulvum* [119], the dominant *Spirochaetaceae* symbionts of *C. rubrum* are likely not maternally transmitted but environmentally acquired [120]. Early work on tropical soft corals (e.g. *Litophyton arboreum, Nephthea* sp., *X. umbellata*) showed that settlement and development of planula larvae were halted by antibiotics, indicating the relevance of associated or environmental bacteria [122]. Congruently, bacterial cues from *Ruegeria* and *Alteromonas* were suggested to promote larval settlement and metamorphosis in *R. fulvum fulvum* [119]. These findings highlight the importance of associated or environmentally acquired symbionts during early octocoral development and settlement. However, the exact microbial taxa, their transmission modes, and the molecular mechanisms underpinning recruitment remain unknown. Future research should prioritize controlled gnotobiotic larval experiments with microbiome manipulation (e.g. antibiotic treatment followed by targeted re-inoculation of native strains), combined with integrated multi-omics analyses, and assessments of host morphology and physiology. Assays explicitly linking octocoral larval settlement, metamorphosis into the primary polyp, and early colony development to ocean change are critically needed. Early life stages represent major bottlenecks in coral life cycles, strongly influencing recruitment success, population persistence, and species distribution. These stages can be particularly sensitive to environmental stressors, which may affect both the benthic habitats where larvae settle and the larvae microbiome.

### Response of the octocoral holobiont to environmental stressors

Although octocoral holobionts are often considered resilient on degraded reefs where scleractinians have been decimated [123], they are still sensitive to climate change. Rising temperatures and other physicochemical shifts in seawater threaten to disrupt octocoral–microbiota associations. Most studies addressing climate change effects on the octocoral holobiont have focused on ocean warming [51, 53, 124–128], whereas the impacts of ocean acidification and deoxygenation remain understudied. Ocean acidification impairs coral calcification and alters marine carbon chemistry in ways that may compromise overall octocoral health and resilience, while deoxygenation reduces ATP production, induces tissue necrosis, and can favor facultatively anaerobic pathogens. However, short-term exposure of *Lobophytum pauciflorum* to decreased pH (7.9), alone or in combination with elevated temperature, did not result in changes in microbial community composition, suggesting a degree of microbiome resilience [129]. This contrasts with the limited number of studies conducted on scleractinians, which reported microbiome destabilization under low pH, with interactive effects of warming and acidification leading to microbial and host dysfunction [130]. Although no studies have investigated deoxygenation effects on octocorals, research on scleractinians indicates that hypoxic conditions rapidly destabilize associated microbiomes through increases in anaerobes and opportunists [131].

#### Shifts in symbiont diversity and abundance

Octocoral bleaching, dysbiosis, and disease outbreaks have all been linked to rising seawater temperatures [126, 128, 132]. Both laboratory-based and field studies during marine heatwaves are improving our understanding of octocoral microbiome dynamics and holobiont resilience. Photosymbiotic octocorals exhibit species-, symbiont-, and genotype-specific responses to thermal stress [7, 17, 26, 133]. During the 2015/2016 El Niño, all octocorals were lost at the Kiritimati Atoll sites studied [134]. Mass bleaching events between 1998 and 2020 also led to a loss of octocorals on the Great Barrier Reef, although with strong taxonomic differences [135]. In contrast, many Caribbean octocorals, particularly those hosting *Breviolum* symbionts, are comparatively resistant [26, 136]. Autotrophic gorgonians with obligate, specialized Symbiodiniaceae associations tend to resist bleaching, whereas more heterotrophic species with facultative photosymbiosis (e.g. some *Pterogorgia*, *Eunicea*, *Plexaura*, *Plexaurella*) are more vulnerable [17]. Thermal stress response of associated Symbiodiniaceae spans from tissue migration (as protective measure) to expulsion and death [26, 133]. Symbiont migration in the gastrovascular lumen is a response to increased reactive oxygen species levels in the tissue, possibly to escape cell death and provide a reservoir of healthy symbionts once conditions return to normal [137]. In some cases, symbiont shuffling occurs: more thermally tolerant, low-abundance symbionts increase in dominance, conferring thermal resistance to the holobiont [26]. In a heat-stress experiment with *Sclerophytum heterospiculatum*, chlorophyll *b* and carotenoid contents increased relative to Symbiodiniaceae proportions, suggesting chlorophyll repackaging, enhanced light absorption, and increased antioxidant capacity as compensatory mechanisms [138]. Symbiodiniaceae of octocorals also appear more resistant than those of scleractinians to organic carbon enrichment [139], or to acidification, in this latter case protected by the thick tissue of octocorals [140]. Far less is known about climate impacts on other microeukaryotes, though few studies have investigated Mediterranean octocorals that lack Symbiodiniaceae [51–53]. The *C. rubrum* eukaryome was shown to respond more rapidly than the associated bacteria to abnormally high temperatures [51, 53]. Stressed microeukaryotic communities show increased species diversity and altered community structure, including reduced relative abundance of dominant taxa such as Labyrinthulomycetes (stramenopiles), Ostreobiaceae (green algae), and Philasterida (ciliates) [51, 53], although the functional consequences of these shifts remain unknown.

Similar to findings in scleractinians, octocoral-associated bacterial communities are also affected by elevated temperatures [51, 53, 124–128]. Heat stress often increases bacterial diversity and alters community composition, although such changes may not be readily detectable following short-term heat exposure [141]*.* Declines in relative abundance of dominant symbionts such as *Endozoicomonadaceae* and *Spirochaetaceae* are commonly observed changes in temperate octocorals [124, 126, 128, 141]. These reductions in relative abundance do not necessarily indicate a loss of mutualistic bacteria, but rather the proliferation of opportunists such as *Vibrionaceae* [126, 128]. Taxon-specific quantitative polymerase chain reaction (qPCR) analyses show that the absolute abundance of *Spirochaetaceae* and *Endozoicomonas* in gorgonians remains stable under heat stress, even as their relative abundance declines [126]. The resulting shifts in microbiome structure are instead driven by the expansion of other opportunists, such as *Vibrio*, which increase by up to 3000-fold in heat-stressed colonies [126]. Correspondingly, a metatranscriptomic study on *C. rubrum* showed that dominant *Spirochaetota* remained functionally stable under thermal stress, continuing to express genes involved in the biosynthesis of essential amino acids and vitamins [142]. However, temperature fluctuations differentially affected other bacterial groups: *Alphaproteobacteria* and *Vibrionales* exhibited opportunistic responses, including increased expression of genes associated with flagellar motility and virulence factors, whereas non-*Vibrionales Gammaproteobacteria* showed reduced functional activity [142].

#### Changes in microbiome functions under dysbiosis

Dysbiosis can lead to the loss of essential symbiont functions and promote temperature-dependent diseases [132] (Fig. 1b). Numerous octocoral disease outbreaks have been linked to temperature-responsive pathogens, including the bacteria *Vibrio coralliilyticus* and *Oscillatoria (Cyanobacteria)*, and the fungi *Aspergillus, Trichoderma, Cladosporium*, and *Penicillium* [132]. In dysbiotic gorgonian microbiomes, genes associated with pathogenicity, virulence, and host invasion, as well as increased arginase and nitric oxide reductase genes, have been detected, suggesting suppression of the host’s nitric oxide defense response (Fig. 1) [29]. Dysbiosis is also characterized by higher levels of secondary metabolite BGCs, and homoserine lactone genes involved in cell density–dependent chemical signaling (quorum sensing) [29]. Virulence factors are frequently regulated by quorum sensing, and the enriched secondary metabolite BGCs observed under dysbiosis likely reflect elevated microbial competition within the pathobiome.

#### Microbiome recovery from stress

The extent of dysbiosis and the ability of the microbiome to return to a state of equilibrium (microbiome resilience) may strongly influence coral health and survival under heat stress. In *C. rubrum*, a short-term moderate (19°C) and severe (23°C) temperature increase altered microbiome composition, but partial recovery occurred within one week after returning to control temperatures (15°C) [51]. Although coral stress-response genes (HSP70 and TNRF) indicated physiological recovery, microbial opportunists such as *Vibrio* persisted at high levels [51], potentially affecting near future responses to recurrent Mediterranean heatwaves. Moreover, work on *G. ventalina* showed that microbiome shifts persisted for up to a year following the 2010 Caribbean thermal anomaly [127]. This suggests that, after recovery, heat-stressed octocorals may shift to a new microbial equilibrium, an alternative stable state (see detailed discussion below), rather than returning to their original state. While different community configurations may represent stable states, they do not necessarily confer equivalent benefits to the host. Evidence from the human gut microbiome indicates that disturbance-driven shifts into alternative stable states can incur fitness costs, including increased disease susceptibility, altered colonization resistance, or antibiotic susceptibility changes [143]. As such, longitudinal monitoring of host health and resilience following stress exposure, recovery, and microbiome shifts will be critical to understanding long-term impacts on octocoral holobiont fitness.

## Conclusions and future directions

Octocorals are attracting renewed attention as some species increase in dominance on degraded reefs, supported by comparatively rapid growth and, in certain taxa, higher thermal tolerance. Octocoral microbiomes tend to be stable and host-specific, providing a strong foundation for studying host–symbiont interactions and coevolution. Host genomic and transcriptomic resources are emerging for several octocoral species, while bioprospecting pipelines for bioactive compounds are already more advanced in octocorals than in scleractinians (Fig. 5). Despite this progress, a mechanistic understanding of octocoral holobiont function remains limited, in part due to persistent taxonomic challenges. Pronounced morphological plasticity, cryptic diversity, and mismatches between morphological and genetic classifications continue to complicate octocoral systematics [1]. Moreover, many genera remain poorly characterized from morphological, ecological, and molecular perspectives, reflecting strong taxonomic and sampling biases across the group. Together, unresolved species complexes, uneven sampling, and limited host taxonomic expertise constrain our ability to robustly identify species-specific microbiome associations and infer coevolutionary dynamics. Nevertheless, important advances are beginning to address these limitations. Improved molecular tools, including the extended octocoral barcode (COI + igR1 + mtMUTS) and integration of voucher-based morphology with whole-genome sequencing, are enhancing species resolution. Large-scale initiatives such as the Aquatic Symbiosis Genome Project [144] are expanding genomic resources for octocoral holobionts, laying foundations for a more holistic understanding of octocoral–microbe interactions from both host and microbiome perspectives. To strengthen future microbiome research, we explicitly advocate for the routine collection and deposition of voucher specimens together with associated host sequence data linked to microbiome datasets, thereby ensuring taxonomic traceability and facilitating reproducibility and comparative analyses across studies.

**Figure 5:**
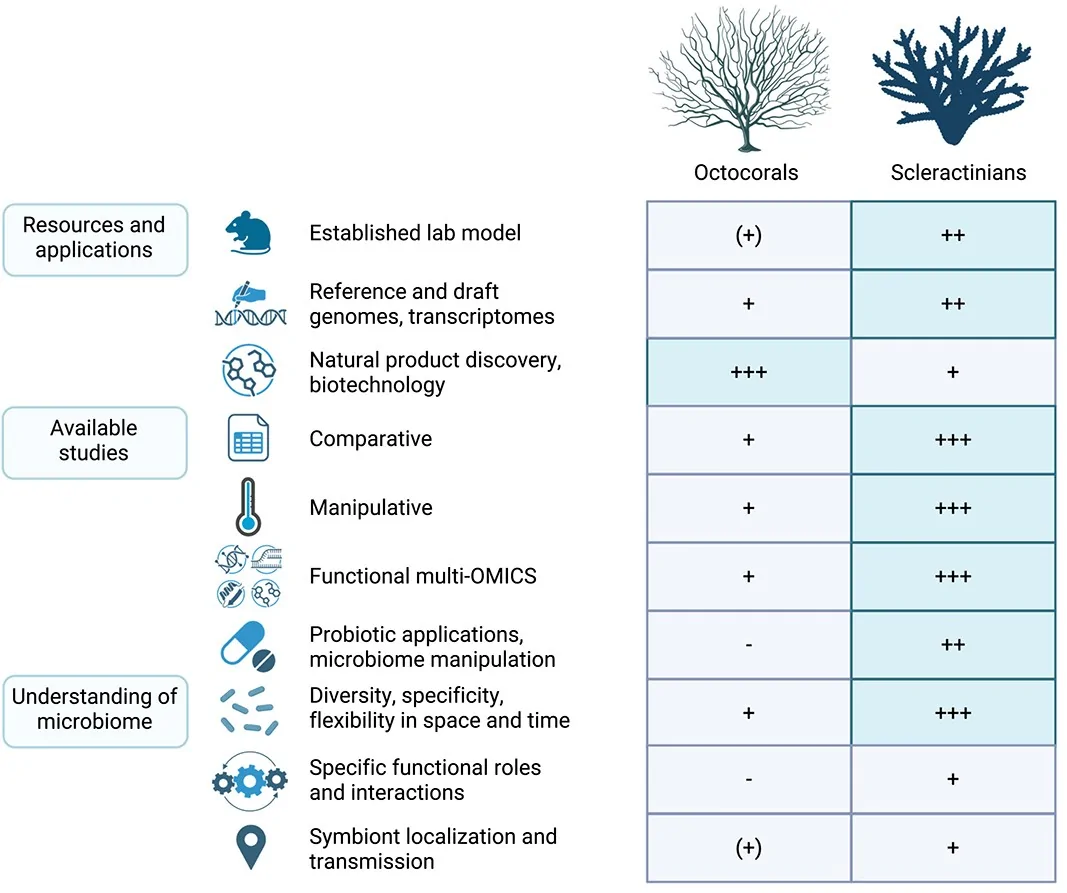
Comparative status of holobiont research in octocorals versus scleractinian corals. Symbols denote the availability of studies and/or current knowledge for the respective field and coral type: +++ = many studies available/solid knowledge; ++ = some studies available; + = few studies available; (+) = isolated study available/very scarce knowledge; − = no studies available. Figure created in BioRender. Pogoreutz, C. (2026) https://BioRender.com/re9sgis.

### Knowledge gaps and emerging technologies to advance octocoral holobiont research

In contrast to well-established scleractinian model systems (e.g. *Stylophora pistillata*, *Pocillopora damicornis*, *Acropora millepora*), standardized culturing systems, and controlled experimental models remain scarce for most octocorals (Fig. 5). Consequently, microbiome studies are dominated by descriptive, diversity-focused surveys, with relatively few functional multi-omics investigations or manipulative experiments linking microbial dynamics to host physiology, stress tolerance and recovery, or disease susceptibility. Moreover, while bacterial associates are increasingly characterized, viruses, fungi, archaea, and non-Symbiodiniaceae protists remain poorly resolved components of the octocoral holobiont, as does their role in holobiont function, resilience, and adaptation. Research has focused predominantly on tropical shallow-water soft corals and Mediterranean and Northeast Atlantic gorgonians. Sea pens, cold-water-, and mesophotic octocorals, often hosting highly conserved microbiomes, are still underrepresented. This taxonomic and ecological bias limits our ability to assess phylosymbiosis, biogeography, and environmental shaping forces among octocoral holobionts. Expanding research across underrepresented host groups and geographic regions will therefore be critical to identify broader ecological and evolutionary patterns.

Even as microbial functional insights become increasingly available, most evidence remains correlative rather than causal. Key processes such as symbiosis establishment, nutrient cycling, and stress mitigation are largely inferred from functional gene content or metabolic rate measurements, while direct links between microbial taxa, their activities, and host fitness remain unresolved. Little is known about how octocorals select, shape, and maintain their microbiota, or the role of the host immune system in modulating microbiome assembly and stability. Likewise, the drivers of alternative microbial equilibria following stress remain a critical gap. In line with alternative stable states theory [145, 146], the octocoral holobiont likely persists in different self-regulating and dynamic host–microbiome configurations resulting from the interplay between host physiology, microbiome composition, and local environmental stressors. Such alternative configurations may explain the high intraspecific variability reported for some octocoral species [29, 46]. How these alternative states differ in system-level functioning, and how they influence future susceptibility to disease and additional environmental stress, remains a key frontier in octocoral research. Repeated stress–recovery experiments that deliberately shift the system into alternative stable states, followed by exposure to subsequent stressors and combined with host physiological and microbiome monitoring, will be essential to assess the potential costs and benefits associated with alternative stable states.

Increasing marine heatwaves and disease events demand mechanistic links between holobiont shifts and host mortality; these links are often missing or inconsistent. Addressing these knowledge gaps will require a transition toward integrated and mechanistic research frameworks. Future studies should combine microbiome manipulation and inoculation experiments with controlled stress assays, host physiological measurements, and multi-omics approaches (e.g. metatranscriptomics, metaproteomics, metabolomics) to directly connect microbial identity and function in hospite. Stable isotope tracing (with e.g. ^13^C-, ^15^N-, or ^34^S-labeled substrates) combined with NanoSIMS technology provides a powerful approach to map *in situ* assimilation and translocation of carbon, nitrogen, or sulfur substrates at high spatial resolution (nanometer scale) within the holobiont. Combined with FISH, isotope enrichment can be assigned to specific microbial taxa, allowing direct linkage between metabolism and microbial identity. This will help resolve metabolic interactions, including competition, cooperation, cross-feeding and nutrient exchange among bacteria, fungi, Symbiodiniaceae, and the host, moving beyond individual components to system-level understanding. Additional emerging technologies such as laser-capture microdissection-coupled metagenomics [147], spatial (meta) transcriptomics [148], and spatial metabolomics using MALDI mass spectrometry imaging and FISH [149] hold considerable promise of linking symbiont localization with region-specific taxonomic and functional profiles and heterogeneity, microbial activities, and niche partitioning within the octocoral holobiont. Future research should likewise prioritize the development of standardized quantitative frameworks for microbial cell enumeration and biomass estimation in octocorals. Broader application of approaches such as flow cytometry and qPCR will be essential to enable robust and comparative assessments of microbial abundances across octocorals versus scleractinian corals.

### Establishing robust octocoral model species

Octocorallia are highly diverse, encompassing two orders and thousands of species with distinct physiological and microbial characteristics. Consequently, no single species is likely to represent the full diversity of octocoral–microbiome interactions. We thus suggest that future efforts should focus on establishing a few robust model species spanning both octocoral orders and collectively representing major morphotypes, biogeographic regions, reproductive-, and trophic strategies, including species with and without photosymbionts. Priority should be given to species that are widespread and accessible, can be maintained under laboratory conditions, and for which foundational ecological and microbiological knowledge is already available. Potential candidates include: a temperate Mediterranean/Atlantic gorgonian brooder, e.g. *P. clavata* (useful model for ocean warming susceptibility in non-photosymbiotic mesophotic octocorals); a tropical western Atlantic sea fan, e.g. *G. ventalina* (a photosymbiotic shallow-water species and potential model for octocoral diseases); an Indo-Pacific soft coral, e.g. *X. umbellata* (useful model for invasiveness and experimental studies); a representative Red Sea soft coral, e.g. *Sclerophytum* (contributor to three-dimensional coral reef frameworks); and a pennatulacean sea pen, e.g. *Pennatula rubra* (broadcast spawner, extending to deep sea habitats). Establishing such octocoral models will require a multimodal framework integrating molecular, physiological, and ecological resources such as well-resolved host taxonomy, availability of host genomes and transcriptomes, characterization of tissue organization and physiology, and knowledge of reproduction, development, and feeding ecology. Such resources will greatly facilitate manipulative, mechanistic investigations of host–microbiome interactions. The same species could also serve as priority targets for probiotic applications, as a stronger understanding of their biology and microbiome dynamics would improve interpretation of experimental readouts. Microbiome-based interventions are indeed an emerging frontier in coral reef research. Coral probiotics have shown promise in enhancing stress tolerance in scleractinians [150, 151], yet no probiotic strategies have been developed or tested for octocorals (Fig. 5). This approach relies on native microorganisms with host-beneficial traits that modulate, maintain, or restore the coral microbiota. The establishment of standardized experimental systems and functional microbial assays will be key prerequisites for the future translation of microbiome-engineering approaches to octocoral restoration.

In conclusion, integrating robust model systems, manipulative experiments, host physiology, multi-omics, and spatially resolved functional approaches is key to identifying microbial functions and metabolic pathways directly linked to host fitness, resilience, and disease resistance under realistic environmental scenarios. Such advances will facilitate the identification of holobiont taxonomic and functional indicators that can be incorporated into early-warning systems for octocoral population decline, analogous to physiological biomarkers (e.g. photosynthetic efficiency) already used in scleractinian coral conservation practice. Shifts in key octocoral-associated microbial taxa or functions can serve as sensitive bio-monitoring indicators of ecosystem stress, while microbiome profiling can assess the health of outplanted octocorals during restoration and guide the application of probiotic consortia, particularly in octocoral nurseries to enhance early-life fitness. By linking microbiome states to host physiological thresholds, these data could improve resilience forecasting by predicting population vulnerability to warming and other environmental stressors. Ultimately, integrating functional microbiome insights into ecological frameworks will provide managers with a predictive toolkit for bio-monitoring, restoration assessment, and rapid decision-making, including prioritizing protection of reefs with resilient microbiome signatures and implementing microbiome-based interventions at stressed octocoral sites. To guide future progress, we summarize below the key priorities for advancing octocoral holobiont research.

### Roadmap for addressing key challenges in octocoral microbiome research

(1) Invest in octocoral taxonomy and preserve voucher specimens to strengthen the links between host taxonomy and microbiome datasets.
(2) Develop tractable model systems by establishing species that can be cultured, experimentally manipulated, and studied across generations.
(3) Experimentally validate the functions of dominant symbionts, such as *Endozoicomonadaceae* and other persistent octocoral associates.
(4) Advance the spatial ecology of the octocoral microbiome by determining where microorganisms reside within host tissues and linking their localization to function through the integration of imaging, genomics, and metabolomics.
(5) Move from correlation to causation through experimental manipulation of microbiomes and by demonstrating microbial contributions to host fitness, stress tolerance, and disease resistance.
(6) Predict taxonomic and/or functional microbiome indicators of resilience by identifying microbial traits that forecast host performance under warming, acidification, deoxygenation, and disease pressure.

## Supplementary Material

Keller-Costa_et_al_Additional_File_1_clean_R2_wrag246

Keller-Costa_et_al_Additional_Tables_S1-S4_Revised_wrag246

## Data Availability

All genomes, MAGs and SAGs explored in this study for meta-analysis are publicly available on NCBI GenBank. Full details on assembly accession numbers, genome metadata and reference to their original publication are available in Additional Table S1. Genome functional annotations (COG, KEGG, BGCs) are available in Additional Tables S2–S4.
