# Supplementary material for: Symbiotic interactions and climate change implications of the octocoral microbiome
Source: ISME J. 2026 Sep 15;20(1):wrag246. doi: 10.1093/ismejo/wrag246 (PMC13688358; doi:10.1093/ismejo/wrag246)
Supplement: Supplementary file 1 [file keller-costa_et_al_additional_file_1_clean_r2_wrag246.docx]

REVIEW ARTICLE

**ADDITIONAL FILE 1 – METHODOLOGY GENOME ANALYSIS**

**TITLE**

**Symbiotic interactions and climate change implications of the octocoral microbiome**

**RUNNING TITLE**

**The octocoral microbiome**

**AUTHORS**

**Keller-Costa, T.**^1,2*^ (ORCID 0000-0003-3702-9192), **Tignat-Perrier, R.**^3,4^ (ORCID 0000-0002-5445-4652), **Marques, M.**^1,2^ (ORCID 0000-0001-9443-0893), **Ferrier-Pagès, C.**^3^ (ORCID 0000-0002-0357-4486), **Pogoreutz, C.** ^5*^ (ORCID 0000-0002-2853-7673)

**AFFILIATION**

^1^Department of Bioengineering, Instituto Superior Técnico, Av. Rovisco Pais 1, 1049-001 Lisbon, Portugal

^2^iBB - Institute for Bioengineering and Biosciences and i4HB - Institute for Health and Bioeconomy, Instituto Superior Técnico, Av. Rovisco Pais 1, 1049-001 Lisbon, Portugal

^3^Equipe Biologie corallienne, Centre Scientifique de Monaco, MC-98000 Monaco

^4^Unité de Recherche sur la Biologie des Coraux Précieux CSM - CHANEL, Centre Scientifique de Monaco, MC-98000 Monaco

^5^UAR 3278 CRIOBE, PSL Université Paris: EPHE-UPVD-CNRS, Université de Perpignan Via Domitia, 52 Avenue Paul Alduy, 66860, Perpignan Cedex, France

***CORRESPONDENCE:** Tina Keller-Costa: Instituto Superior Técnico, Av. Rovisco Pais 1, 1049-001 Lisbon, [tinakellercosta@tecnico.ulisboa.pt](mailto:tinakellercosta@tecnico.ulisboa.pt); Claudia Pogoreutz: [claudia.pogoreutz@univ-perp.fr](mailto:claudia.pogoreutz@univ-perp.fr)

**KEYWORDS**

Holobiont, host-microbe interactions, symbioses, soft corals, gorgonians, *Endozoicomonadaceae*, *Spirochaetaceae*, nutrient cycling, pathobiome*,* ocean warming

**METHODS**

**Retrieval of octocoral-associated bacterial genomes**

Genomes (assembly files in .fasta format) of octocoral-associated prokaryotes were retrieved from the National Center of Biotechnology Information (NCBI GenBank [1], last accessed 08.09.2025), comprising 39 isolate genomes, 39 metagenome-assembled genomes (MAGs), and one single-amplified genome (SAG). The NCBI databases *BioProject, BioSample, Genome,* and *Assembly* were queried using the search terms “Bacteria” OR “Archaea” AND “Octocoral” OR “Gorgonian” OR “Soft Coral” OR “Sea Fan” OR “Sea Whip”, resulting in 79 bacterial genomes. Genome metadata, including genome type, bacterial taxonomy, octocoral host species and health status, geographic origin, and sequencing technology, were retrieved from the corresponding *BioProject*, *BioSample*, or *Assembly* records in NCBI. When necessary, missing information was supplemented by consulting the associated publications. Genomes were uploaded on the Department of Energy (DOE) Systems Biology Knowledgebase (KBase) online platform ([www.kbase.us](http://www.kbase.us)) [2], where they were assessed for their completeness and contamination with CheckM1 (v1.0.18) [3]. All genomes had >60% completeness, <5% contamination, and an overall quality score (completeness – 5× contamination) >50%. All genomes had their GC content and genome size retrieved from the respective assembly files on KBase. General genome features and the number of coding sequences (CDS) were obtained using Prokka (v.1.14.5) [4] within KBase. Detailed information on the assembly accession numbers, genome metrics, and metadata for all genomes analysed in this study is provided in **Additional Table S1**.

**Phylogenomic analysis**

A phylogenomic tree was constructed for the 79 octocoral-associated bacterial genomes using the SpeciesTreeBuilder (v0.1.4) application with the function *Insert Set of Genomes into Species Tree* of the KBase online platform [2]. Genomes were previously annotated with Prokka (v1.14.5) [4] and used as input within KBase. Sequences were aligned with a multiple sequence alignment based on a set of 49 core Clusters of Orthologous Groups (COGs) of protein [2]. The FastTree2 algorithm [5] was used with the maximum likelihood (ML) method and Jukes–Cantor evolutionary model with a CAT approximation to generate a tree with 1000 repetitions to estimate bootstrap support values [5]. The phylogenomic tree was then styled in iTOL v7 [6] and refined in Inkscape (https://inkscape.org/).

**KEGG annotation, level of completeness of KEGG modules, and heatmap construction**

Using *Anvi’o v8* (<https://github.com/merenlab/anvio>; [7]), the 79 genomes, MAGs and SAGs were processed as external genomes. For each genome/MAG/SAG, the following commands were run with default parameters: *anvi-script-reformat-fasta*, *anvi-gen-contigs-database*, *anvi-run-hmms*, *anvi-run-kegg-kofams*, and *anvi-estimate-metabolism* (with the option --matrix-format).
The commands *anvi-script-reformat-fasta* and *anvi-gen-contigs-database* reformatted the FASTA files, removed contigs <1000 bp, predicted open reading frames in prokaryotic contigs using Prodigal v2.6.3 [8], and generated an anvi’o contigs database. Subsequently, commands *anvi-run-hmms* and *anvi-run-kegg-kofams* were executed to search for Hidden Markov Models (HMMs) using HMMER v3.3.2 [9] across all contig databases, and annotate genes and metabolic pathways using the Kyoto Encyclopedia of Genes and Genomes (KEGG) database. Finally, the *anvi-estimate-metabolism* command was used to identify KEGG modules for each genome/MAG/SAG and to generate completeness and presence–absence matrices. KEGG modules related to nitrogen-, carbon-, and sulfur metabolism, amino acid biosynthesis, cofactor and vitamin metabolism were selected for heatmap construction (**Additional Table S3**). When multiple KEGG modules corresponded to the same biosynthetic pathway, the module with the highest completeness value was retained. The heatmap was then generated in *R* (v4.5.1) using the packages *reshape2* [10] and *gplots* (function heatmap.2; [11]).

**Predicted oxygen tolerance**

The software package GenomeSPOT was used with default parameters to predict phenotypic traits from genomic sequences, with particular focus on predicted oxygen tolerance, for the 79 octocoral-associated bacterial genomes [12].

**COG annotation for chitinase detection**

Clusters of Orthologous Groups of proteins (COG) annotation of the 79 octocoral-associated bacterial genomes, including screening for chitinase-encoding genes, was performed using the automated genome annotation pipeline Melange, as documented on GitHub (<https://sandragodinhosilva.github.io/MeLanGE>) and earlier described [13]. Complete COG profiles of the 79 octocoral-associated bacterial genomes are provided in **Additional Table S2**.

**Annotation of biosynthetic gene clusters (BGCs)**

Biosynthetic gene clusters (BGCs) encoding secondary metabolites were annotated with antiSMASH v8.0.4 [14] via the multiSMASH wrapper (10.5281/zenodo.8276143) with default parameters. Complete antiSMASH annotations for the 79 octocoral-associated bacterial genomes are provided in **Additional Table S4**.

**REFERENCES**

1.	Sayers EW, Bolton EE, Brister JR, et al. Database resources of the national center for biotechnology information. *Nucleic Acids Reseach*. 2022;50(D1):D20-d26. doi:10.1093/nar/gkab1112

2.	Arkin AP, Cottingham RW, Henry CS, et al. KBase: The United States Department of Energy Systems Biology Knowledgebase. *Nature Biotechnology*. 2018;36(7):566-569. doi:10.1038/nbt.4163

3.	Parks DH, Imelfort M, Skennerton CT, Hugenholtz P, Tyson GW. CheckM: assessing the quality of microbial genomes recovered from isolates, single cells, and metagenomes. *Genome Research*. 2015;25(7):1043-55. doi:10.1101/gr.186072.114

4.	Seemann T. Prokka: rapid prokaryotic genome annotation. *Bioinformatics*. 2014;30(14):2068-2069. doi:10.1093/bioinformatics/btu153

5.	Price MN, Dehal PS, Arkin AP. FastTree 2 - approximately maximum-likelihood trees for large alignments. *PloS One*. 2010;5(3):e9490-e9490. doi:10.1371/journal.pone.0009490

6.	Letunic I, Bork P. Interactive Tree of Life (iTOL) v6: recent updates to the phylogenetic tree display and annotation tool. *Nucleic Acids Research*. 2024;52(W1):W78-w82. doi:10.1093/nar/gkae268

7.	Eren AM, Kiefl E, Shaiber A, et al. Community-led, integrated, reproducible multi-omics with anvi'o. *Nature Microbiology*. 2021;6(1):3-6. doi:10.1038/s41564-020-00834-3

8.	Hyatt D, Chen GL, Locascio PF, Land ML, Larimer FW, Hauser LJ. Prodigal: prokaryotic gene recognition and translation initiation site identification. *BMC Bioinformatics*. 2010;11:119. doi:10.1186/1471-2105-11-119

9.	Eddy SR. Accelerated profile HMM searches. *PLoS computational biology*. 2011;7(10):e1002195. doi:10.1371/journal.pcbi.1002195

10.	Wickham H. Reshaping data with the reshape package. *Journal of Statistical Software*. 2007;21(12):1-20.

11.	*gplots: Various R programming tools for plotting data. R package version 3.2.0,* [*https://github.com/talgalili/gplots*](https://github.com/talgalili/gplots). 2025.

12.	Barnum TP, Crits-Christoph A, Molla M, Carini P, Lee HH, Ostrov N. Predicting microbial growth conditions from amino acid composition. *bioRxiv*. 2024:2024.03.22.586313. doi:10.1101/2024.03.22.586313

13.	Keller-Costa T, Kozma L, Silva SG, et al. Metagenomics-resolved genomics provides novel insights into chitin turnover, metabolic specialization, and niche partitioning in the octocoral microbiome. *Microbiome*. 2022;10(1):151. doi:10.1186/s40168-022-01343-7

14.	Blin K, Shaw S, Vader L, et al. antiSMASH 8.0: extended gene cluster detection capabilities and analyses of chemistry, enzymology, and regulation. *Nucleic Acids Research*. 2025;53(W1):W32-w38. doi:10.1093/nar/gkaf334
